# IL-33 and IL-33-derived DC-based tumor immunotherapy

**DOI:** 10.1038/s12276-024-01249-4

**Published:** 2024-06-03

**Authors:** Myeong-Ho Kang, Yong-Soo Bae

**Affiliations:** 1 Department of Biological Sciences, Sungkyunkwan University, 2066 Seobu-ro, Suwon, Gyeonggi-do 16419 Republic of Korea; 2 Center for Immune Research on Non-Lymphoid Organs, Sungkyunkwan University, 2066 Seobu-ro, Suwon, Gyeonggi-do 16419 Republic of Korea

**Keywords:** Immunization, Conventional dendritic cells

## Abstract

Interleukin-33 (IL-33), a member of the IL-1 family, is a cytokine released in response to tissue damage and is recognized as an alarmin. The multifaceted roles of IL-33 in tumor progression have sparked controversy within the scientific community. However, most findings generally indicate that endogenous IL-33 has a protumor effect, while exogenous IL-33 often has an antitumor effect in most cases. This review covers the general characteristics of IL-33 and its effects on tumor growth, with detailed information on the immunological mechanisms associated with dendritic cells (DCs). Notably, DCs possess the capability to uptake, process, and present antigens to CD8^+^ T cells, positioning them as professional antigen-presenting cells. Recent findings from our research highlight the direct association between the tumor-suppressive effects of exogenous IL-33 and a novel subset of highly immunogenic cDC1s. Exogenous IL-33 induces the development of these highly immunogenic cDC1s through the activation of other ST2^+^ immune cells both in vivo and in vitro. Recognizing the pivotal role of the immunogenicity of DC vaccines in DC-based tumor immunotherapy, we propose compelling methods to enhance this immunogenicity through the addition of IL-33 and the promotion of highly immunogenic DC generation.

## Exogenous IL-33: Unveiling Tumor-Suppressive Effects on cDC1s

Interleukin-33 (IL-33), a protein that helps the body in combating infections, plays a double role in cancer growth, says a study by Kang and Bae. The research found that IL-33 can both help and hinder cancer growth, contingent upon its concentration and the specific infection-fighting (immune?) cells it interacts with. The study, done on mice, showed that lower concentrations of IL-33 facilitate cancer grow by increasing certain infection-fighting (immune?) cell groups and causing cell multiplication. Conversely, higher concentrations of IL-33 counteract these effects, triggering anti-cancer responses by activating other infection-fighting (immune?) cells. The researchers concluded that IL-33 holds potential for cancer therapy, either through direct injection or by fostering the production of potent infection-fighting (immune?) cells for vaccination. Future research will delve into the implications of these findings for human cancer treatment. "This summary was initially drafted using artificial intelligence, then revised and fact-checked by the author."

## General characteristics of IL-33

**Fig. 1:**
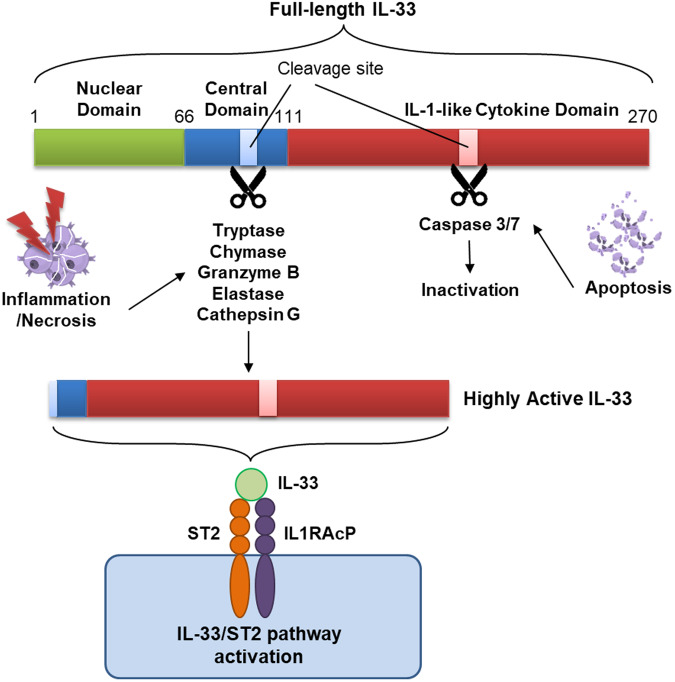
Cleavage of full-length IL-33 and activity. Full-length IL-33, which contains two cleavage sites, is largely composed of a nuclear domain, a central domain, and an IL-1-like cytokine domain. The activity of IL-33 is contingent on biological events. During apoptosis, caspase-3 and -7 are produced and released. These proteins cleave the cleavage site on the IL-1-like cytokine domain, leading to the inactivation of IL-33. Inflammation or necrosis can induce tryptase, chymase, and granzyme B release from neutrophils or elastase and cathepsin G release from mast cells. These inflammatory or necrotic enzymes cleave the cleavage site on the central domain, inducing the activation of IL-33. When active, IL-33 binds to the receptor ST2/IL-1AcP, activating the IL-33/ST2 signaling pathway.

Interleukin-33 (IL-33), sometimes known as a nuclear factor (NF-HEV), is a alarmin cytokine that is released by endothelial cells and epithelial cells in response to danger stimuli^1,2^. Due to its structural similarity, IL-33 is included in the IL-1 family and was also named IL-1F11 following the nomenclature of the IL-1 family^3^. Like IL-1β and IL-18, IL-33 is synthesized in its full-length form (human: 270 aa, mouse: 266 aa), which comprises a nuclear domain, central domain, and IL-1-like domain^2,4^. IL-33 activity is contingent on its cleavage (Fig. 1). During apoptosis, caspase-3 and -7 cleave the caspase site in the IL-1 cytokine domain, leading to the inactivation of IL-33^5,6^. Conversely, inflammatory or necrotic enzymes released from neutrophils and mast cells or allergen-derived enzymes cleave the central domain of IL-33^5^. The cleaved forms of IL-33 produced by these enzymes are approximately 30-fold more active than the full-length form^4,5,7^. IL-33 signaling occurs through the interleukin 1 receptor-like 1 (IL1RL1) receptor, ST2, with the coreceptor IL-1RAcP^3,8,9^. IL-33/ST2 signals primarily act on T helper 2 cells, type 2 innate lymphoid cells (ILC2s), basophils, eosinophils, and mast cells, leading to the secretion of type 2 cytokines such as IL-4, IL-5, and IL-13, which are associated with allergic inflammation^3,4^. However, the role of IL-33 is not limited to the activation of type 2 immunity. Several studies have revealed that IL-33 is also involved in activating various cells, including CD8^+^ T cells, NK cells, neutrophils, macrophages, B cells, NKT cells, regulatory T cells (Tregs), and dendritic cells (DCs)^5^. This broad involvement of IL-33 in various immune cells underscores its potential impact on nonallergic diseases, such as cancer.

## Controversial role of IL-33 in cancer

The immunogenicity of IL-33 is contingent on the specific immune cells stimulated or the quantity of IL-33. Given the diverse involvement of IL-33, its roles in cancer remain controversial^10,11^. The impact of IL-33 on tumors varies depending on its source and concentration (Table 1). In studies of IL-33 in the context of cancer, *Il33*^−/−^ mice and *Il1rl1*^−/−^ mice have been shown to have greater tumor growth. Moreover, the administration of IL-33-blocking antibodies or soluble ST2 has been shown to have protumoral effects. These studies have demonstrated that endogenous IL-33 is associated with immune responses to tumors. However, previous studies have shown that the administration of exogenous IL-33 and IL-33 overexpression have antitumor effects. The concentration of endogenous IL-33 in mouse tissue or fluid is several ng/g of tissue or ng/ml of fluid^12,13^. However, when IL-33 is overexpressed in tumor cells or is directly administered, its concentration ranges from tens of ng to several μg^14^. In cholangiocarcinoma, high-dose IL-33 inhibits cell migration, while low-dose IL-33 promotes cell migration^15^. This means that tumoral responses to IL-33 are likely contingent on its concentration.

**Table 1:** Controversial roles of IL-33 in cancer.

Pro-tumor effect				
Target cell	Type of tumor	Origin of IL-33	Verification	Refs.
Treg	Lung tumor	Endogenous	ST2^−/−^ mice Soluble ST2	^18,20^
	Intestinal tumor	Endogenous	ST2^−/−^ mice	^19^
	Melanoma	Endogenous	IL33^−/−^ mice	^21^
Mast cell	Gastric cancer	Endogenous	*gp130*^*FF*^;ST2^−/−^ mice IL-33 blocking antibody	^22,23^
Macrophage	Pancreatic cancer	Endogenous	IL33^−/−^ or ST2^−/−^ mice Soluble ST2	^25^
	Squamous cell carcinoma	Endogenous	IL-33-knockdown	^26^
Tumor cell	Colorectal cancer	Endogenous	IL-33-knockdown	^27^
	Gastric cancer	Endogenous	IL33^−/−^ mice	^28^

Anti-tumor effect				
Eosinophil	Melanoma	Exogenous	Ex vivo treatment	^33^
		Exogenous	Tumoral overexpression	^34^
	Metastatic lung tumor	Exogenous	In vivo injection (i.n)	^35^
Basophil	Melanoma	Exogenous	Ex vivo treatment	^36^
ILC2	Pancreatic cancer	Endogenous	IL33^−/−^ mice, ILC2-depleted mice	^39^
CD8^+^ T cell	Cervical cancer	Exogenous	DNA vaccine adjuvant	^32^
	Melanoma Liver tumor	Exogenous	Tumoral overexpression	^30,37^
	Colon cancer	Endogenous	IL33^−/−^ mice	^40^
CD11b^+^ DCs	Melanoma	Exogenous	In vivo injection (i.p)	^14,43^
	Lung tumor	Exogenous	In vivo injection (i.p)	^42,44^
cDC1	Pancreatic cancer	Exogenous	Batf3^−/−^ mice In vivo injection (i.p)	^39^
	Lymphoma	Exogenous		^31^
	Melanoma	Exogenous	Batf3^−/−^ mice Tumoral overexpression	^41^

### Pro-tumor effect of IL-33

Previously, it was reported that the IL-33/ST2 axis directly expands the Treg population in the intestine and that IL-33-induced IL-2 production by CD11c^+^ cells increases the number of ST2+ Tregs^16,17^. ST2 deficiency in tumor models decreases the Treg population and reduces tumor growth^18,19^. The IL-33/ST2 axis alters the Treg population, thus promoting tumor progression^19,20^. In addition, Treg-intrinsic IL-33 is essential for Treg stability to evade anti-tumor immunity^21^. According to recent studies, IL-33-activated mast cells, which secrete IL-2, also expand ICOS^+^ Tregs to promote gastric cancer^22^. In spontaneous gastric adenoma models, tumor cell-derived IL-33 activates ST2^+^ mast cells^23^. Several factors released from activated mast cells attract tumor-associated macrophages (TAMs), which promote tumor growth and angiogenesis^23^. IL-33/ST2 can stimulate M2 macrophage polarization, which contributes to wound healing^5,24^. In the tumor environment, endogenous IL-33 contributes to the recruitment and M2 polarization of TAMs, which secrete MMP9 or TGF-β to promote cancer progression and metastasis^25,26^. The protumoral effect of IL-33 is not limited to immune cells, as IL-33 can directly induce the proliferation or migration of tumor-associated cells^27,28^. Most of these studies were conducted from the perspective of endogenous IL-33.

### Anti-tumor effect of IL-33

In contrast to earlier reports, several studies have indicated that tumoral overexpression of IL-33 or IL-33 injection can promote antitumor immunity. Similar to endogenous IL-33, exogenous IL-33 has been shown to increase the number of suppressive myeloid cells and Tregs^29–31^. However, exogenous IL-33 overcomes such immunosuppressive effects by activating several immune cells associated with antitumor responses. The mechanisms underlying IL-33-mediated antitumor immune responses are detailed below.

The efficient suppression of tumor growth is observed when IL-33 is used as an adjuvant^32^. IL-33 is well known for stimulating eosinophils, basophils, and ILC2s involved in type 2 immunity. IL-33-activated eosinophils express more effector molecules (ECP, EPX, and granzyme-B), as well as the CD11b/CD18 immune synapse, leading to the killing of tumor cells in vitro and in vivo^33^. In the presence of DPP4 (dipeptidyl peptidase-4) inhibitors, known as antidiabetic drugs, tumoral IL-33 expression increases, promoting eosinophil-mediated antitumor responses^34^. In addition, intranasal administration of IL-33 recruits eosinophils to the lung, preventing the onset of pulmonary metastasis^35^. Like eosinophils, IL-33-activated basophils increase the expression of degranulating molecules such as CD63 and granzyme-B, resulting in tumoricidal properties in vitro^36^. In a recent study, soluble factors from IL-33-activated basophils were shown to induce immunogenic DC differentiation, contributing to the antitumor response^31^. Immunization with IL-33 isoform plasmids enhances antigen-specific CD8^+^ T-cell responses, such as strong CD62L^−^KLRG1^+^ effector–memory CD8^+^ T-cell responses, which contribute to the suppression of tumor growth^32^. Moreover, CD8^+^ T-cell activity was enhanced in a tumor model using cell lines overexpressing IL-33^30,37^. In in vitro CD8^+^ T-cell culture, IL-33 promoted T-cell activation and IFN-γ expression, which suppressed tumor growth^37^. Exogenous IL-33 also contributes to inhibiting tumor growth by overcoming IL-33-mediated Treg expansion^38^. Some reports suggest that endogenous IL-33 can also induce antitumor immunity by overcoming IL-33-induced Treg expansion or boosting tumor-infiltrating ILC2s^39,40^. This finding indicates that exogenous IL-33 suppresses tumor progression in most studies. Several studies have reported the involvement of antigen-presenting cells (APCs), such as DCs, in IL-33-induced antitumor immunity^14,31,39,41–44^. Next, we will review the role of IL-33 in DCs and its potential as a DC-based tumor immunotherapy.

## IL-33 exerts an antitumor effect by enhancing DC immunogenicity

**Fig. 2:**
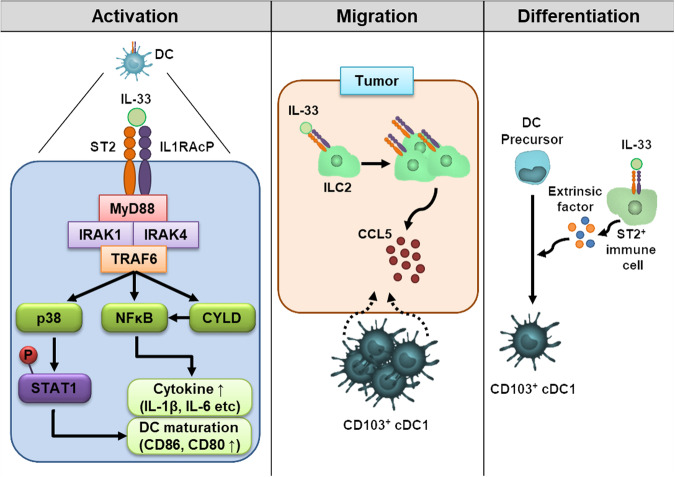
Effects of IL-33 on dendritic cells (DCs). IL-33 affects DCs through three distinct mechanisms: activation, migration, and differentiation. Initially, the binding of IL-33 to ST2/IL1RAcP on DCs triggers the recruitment of MyD88, IRAK1, IRAK4, and TRAF6, which form a molecular complex. This molecular complex is implicated in p38 activation and either direct or CYLD-mediated NF-κB activation. Activated p38 induces STAT1 phosphorylation, leading to DC maturation for CD8^+^ T-cell priming. Concurrently, NF-κB activation promotes DC maturation and inflammatory cytokine release. Second, CCL5, which is secreted from IL-33-expanded ILC2s, promotes the migration of CD103^+^ DCs into tumors. The migrated CD103^+^ cDC1s activate CD8^+^ T cells, fostering antitumor immunity. Finally, IL-33-primed ST2^+^ bystander immune cells secrete soluble factors, leading to the differentiation of DC precursors into CD103^+^ cDC1s.

DCs play a crucial role in priming effector CD8^+^ T cells against tumors through the uptake and presentation of tumor-specific and/or tumor-associated antigens (TSA/TAAs) to antigen-specific T cells^45–49^. Given their pivotal role, DCs represent an intriguing target for tumor immunotherapy^45–49^. Thus, enhancing the immunogenicity and efficacy of DCs is vital for the success of DC-based tumor immunotherapy^47,48^. Previously, the possibility of using IL-33 as an adjuvant was mentioned, as IL-33 activates and recruits APCs^32,50^. Adjuvants act on APCs and enhance antigen presentation^51^. However, among APCs, macrophages exposed to IL-33 can differentiate into protumoral M2 macrophages, and IL-33 induces IL-10-producing regulatory B cells^24,52^. Since only DCs among APCs have the potential for enhanced immunogenicity by IL-33, IL-33 has emerged as a promising candidate for successful DC-based tumor immunotherapy. A previous study reported that ST2 is also expressed in GM-CSF-derived bone marrow-derived DCs (GM-BMDCs) generated in vitro, as determined by intracellular staining with a specific α-ST2 antibody clone (DJ8)^50^. Although ST2 was detected only intracellularly, upon binding of IL-33, ST2 on GM-BMDCs interacts with IL-1RAcP to form a heterodimeric complex^3^, initiating a signaling pathway, as shown in Fig. 2. This complex recruits myeloid differentiation primary response protein 88 (MyD88), IL-1 receptor-associated kinases (IRAK1 and IRAK4), and TNF receptor associated factor 6 (TRAF6)^3^, leading to the activation of the mitogen-activated protein kinase (MAPK) and nuclear factor-κB (NF-κB) pathways^53^. Activated p38 MAPK is known to phosphorylate STAT1^54^, and STAT1 phosphorylation in CD11b^+^ DCs, known as cDC2s, is crucial for the induction of antigen-specific CD8^+^ T-cell priming against tumors^14^. Activation of the NF-κB pathway in CD11b^+^ DCs induces inflammatory cytokines (IL-1β, IL-6, TNF, IL-12, *etc*.) and costimulatory molecules (CD86, CD80, CD40, etc.)^42,55^. The IL-33/ST2/MyD88 axis increases the expression of cylindromatosis (CYLD), a deubiquitination enzyme involved in IL-33-mediated CD11b^+^ DC maturation in pulmonary adenocarcinoma (PA)^42^. MyD88-induced CYLD in CD11b^+^ DCs functions as a tumor suppressor in PA via the regulation of DC maturation and function through the NF-κB signaling pathway^56^. In MyD88-deficient mice, IL-33-mediated DC maturation, T-cell priming, and STAT1 phosphorylation were not observed, and tumor growth was not suppressed by IL-33^14,44^. This means that exogenous IL-33 induces ST2-MyD88-dependent DC activation, which enhances antitumor immunity. In plasmacytoid DCs (pDCs), the expression of the receptor ST2 has been reported, and the IL-33/ST2 axis in pDCs has been shown to inhibit TLR7-mediated activation and production of type I interferon^57,58^. However, the effect of IL-33/ST2 signaling on pDCs in tumors remains unknown.

In the context of GM-BMDC-based tumor immunotherapy, IL-33 administration reportedly drives the induction of IL-9-producing CD8^+^ T cells, potentiating the inhibition of tumor growth^43^. This effect may also be derived from the IL-33/ST2-MyD88 pathway. Treating terminally differentiated DCs with IL-33 may be a viable approach for generating an immunogenic DC vaccine against tumors. Notably, although most of the DCs mentioned thus far are CD11b^+^ cDC2s, DC-based IL-33-induced antitumor immunity is not limited to CD11b^+^ cDC2s.

## IL-33 mediates immunogenic cDC1 differentiation

**Fig. 3:**
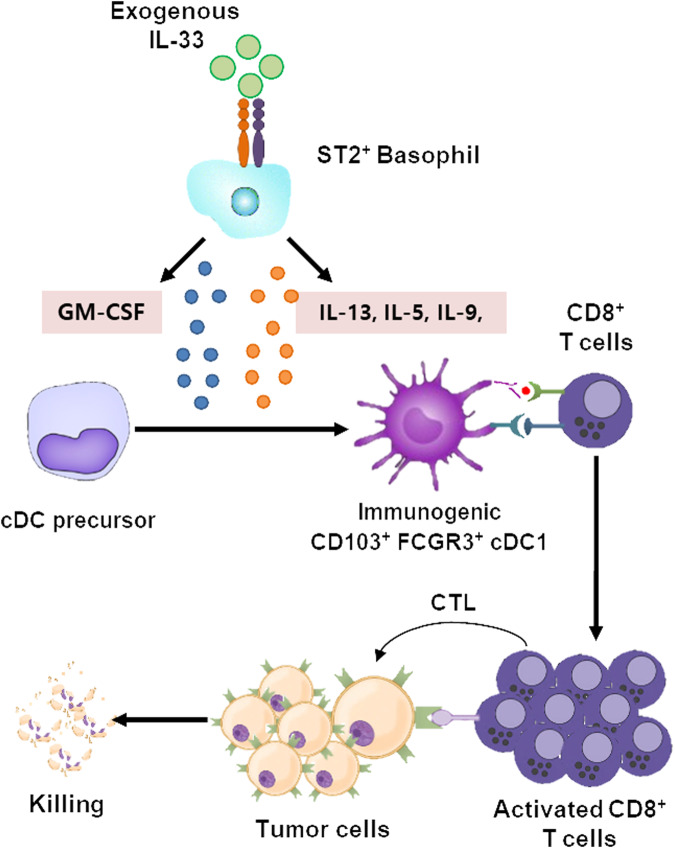
The development of highly immunogenic IL-33-derived CD103^+^ cDC1s and the induction of antitumor immunity. Exogenous IL-33 binds to ST2^+^ basophils, initiating the secretion of IL-33-induced basophil cytokines such as GM-CSF, IL-13, IL-9 and IL-5. These cytokines influence cDC precursors, promoting their differentiation into highly immunogenic FCGR3^+^CD103^+^ cDC1s. This population of highly immunogenic FCGR3^+^CD103^+^ cDC1s facilitates the proliferation and activation of CD8^+^ T cells, leading to the killing of tumor cells via cytotoxic T lymphocyte (CTL) responses.

**Fig. 4:**
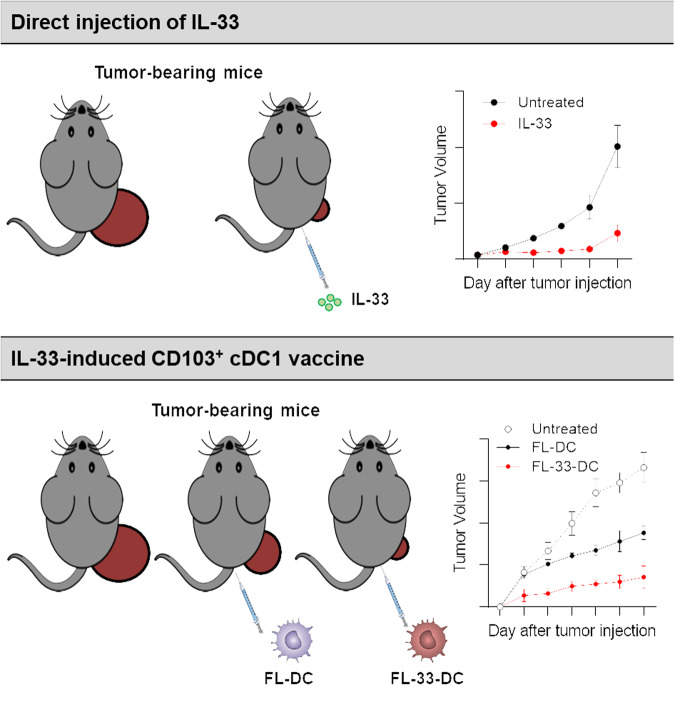
Methods of tumor immunotherapy with recombinant IL-33 or IL-33-derived CD103^+^ cDC1s. Tumor growth is significantly suppressed in mouse tumor models through either direct administration of IL-33 or vaccination with IL-33-induced CD103^+^ cDC1s. Intraperitoneal injection of IL-33 into tumor-bearing mice results in a substantial decrease in tumor growth. Alternatively, vaccination with IL-33-induced CD103^+^ cDC1s derived from Flt3L-BMDCs is another effective method. IL-33-induced CD103^+^ cDC1s demonstrate more potent tumor suppression than conventional DC vaccines.

Several studies have indicated that antitumor responses caused by tumoral IL-33 overexpression or IL-33 administration do not suppress tumor growth in the absence of cDC1s in Batf3^−/−^ mice^31,39,41^. This finding implies that IL-33-mediated antitumor immunity is contingent on cDC1s. In melanoma or pancreatic cancer models, tumoral or recombinant IL-33 increases CD103^+^ DCs, which activate CD8^+^ T-cell responses required for antitumor responses^39,41^. The increase in the number of CD103^+^ DCs in the spleen induced by the injection of IL-33 can be elucidated from the perspectives of migration and/or differentiation of DC precursors. It is well known that the ILC2 population is the major target of IL-33^59^. IL-33-induced migration of CD103^+^ DCs into tumors is orchestrated by CCL5 secreted from IL-33-expanded ILC2s (Fig. 2)^39^. CCL5 is a well-known chemoattractant for cDC1s^60^. The recruited CD103^+^ cDC1s activate CD8^+^ T cells, fostering therapeutic immunity against pancreatic cancer^39^. In contrast to the migration perspective, a recent study reported that IL-33 mediated CD103^+^ cDC1 differentiation^31^. IL-33 injection in vivo or IL-33 treatment of Flt3L-generated BMDCs (FL-BMDCs) increases CD103^+^ cDC1s in an ST2-dependent manner, although ST2 is not expressed in any DC precursors^31^. Soluble factors secreted from ST2^+^ bystander immune cells enable the differentiation of CD103^+^ cDC1s (Fig. 2), and the bystander immune cells were identified as ST2^+^ basophils^31^. Although GM-CSF alone also induces CD103^+^ cDC1s upon in vivo injection or treatment of FL-BMDCs^61^, it does not inhibit tumor growth, unlike IL-33-induced tumor suppression in lymphoma or melanoma models^31^. IL-33-induced CD103^+^ cDC1s exhibit greater immunogenicity, as indicated by the expression of a distinct marker, FCGR3^31^. Among the soluble factors produced by IL-33-activated basophils, GM-CSF induces the expression of CD103 on cDC1 precursors, and the cytokines IL-13, IL-9 and IL-5 are involved in the expression of FCGR3^31^. These cytokines derived from IL-33-activated basophils are essential for the generation of FCGR3^+^CD103^+^ cDC1s, which prime tumor-specific CD8^+^ T cells^31^. FCGR3^+^CD103^+^ cDC1-primed CD8^+^ T cells exhibit robust cytotoxic T lymphocyte (CTL) responses, promoting antitumor immunity (Fig. 3). In tumor immunotherapy utilizing these IL-33-induced FCGR3^+^CD103^+^ cDC1s, tumor growth in solid tumor models or lung metastasis tumor models is significantly suppressed compared to that in control cDC1s or GM-CSF-induced CD103^+^ cDC1s^31^. Thus, both direct IL-33 injection and immunotherapy with IL-33-induced FCGR3^+^CD103^+^ cDC1s increase antitumor immunity in mouse tumor models (Fig. 4).

## Application of IL-33 to human DC-based tumor immunotherapy

**Fig. 5:**
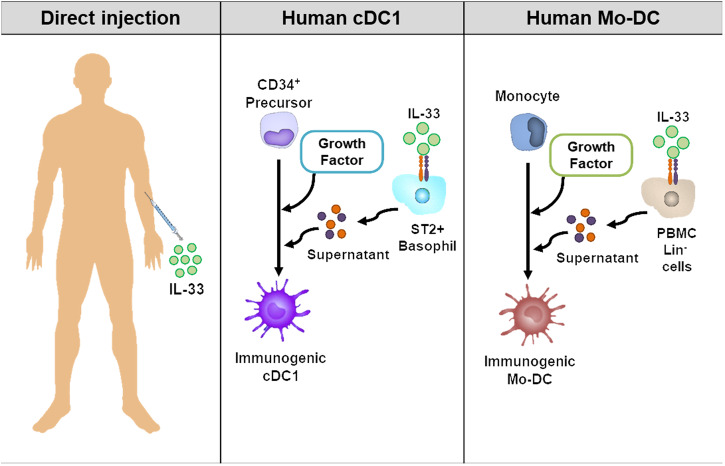
Human application of IL-33 for immunogenic DC generation. Three approaches have been proposed to translate the antitumor effect of IL-33 observed in mice to humans. First, IL-33 could be directly injected into patients with tumors via appropriate routes and at appropriate doses. Second, the generation of human immunogenic cDC1s from CD34^+^ hematopoietic progenitor cells could be considered using IL-33-derived factors from basophils or other immune cells. Finally, IL-33-induced factors from T/B/NK/monocyte-depleted human PBMCs could be employed for the generation of highly immunogenic human monocyte-derived DCs (Mo-DCs).

Based on the effect of IL-33 or IL-33-induced FCGR3^+^CD103^+^ cDC1s on tumor therapy in mice, we propose several approaches to translate the antitumor effect of IL-33 observed in mice to humans (Fig. 5). First, direct injection of IL-33 into cancer patients could be considered. Although there are currently no cases of direct administration of IL-33, it may be plausible to generate highly immunogenic cDC1s, thereby eliciting more potent antitumor responses. IL-33-induced Treg expansion or excessive inflammation should be considered, but these issues could be mitigated by determining the appropriate dose and route. Second, human immunogenic cDC1s can be generated from CD34^+^ hematopoietic progenitor cells using IL-33-derived factors from basophils or other immune cells. While this approach is theoretically ideal, the methods for cDC1 generation and purification for the preparation of DC vaccines are not straightforward due to the prolonged duration and inadequate quantity^62,63^. Finally, highly immunogenic human monocyte-derived DCs (hMo-DCs) can be generated using IL-33-induced factors. ST2 expression is not detected in monocytes or hMo-DCs. However, T/B/NK/monocyte-depleted human peripheral blood mononuclear cells (PBMCs) express ST2 and secrete soluble factors into culture supernatants when cultured in the presence of IL-33^31^. It has been reported that these soluble factors in culture supernatants can generate highly immunogenic human Mo-DCs when administered during the generation of Mo-DCs, and these Mo-DCs are reportedly more potent than control Mo-DCs in priming CD8^+^ T cells^31^. Considering that cryopreserved Mo-DCs maintain their immunogenicity^64^, IL-33-derived highly immunogenic Mo-DCs could be attractive candidates for next-generation DC vaccines. In conclusion, a method for enhancing in vivo immunogenic cDC1s through the direct injection of IL-33 or immunotherapy with IL-33-induced highly immunogenic Mo-DC vaccines may shed light on DC-based tumor immunotherapy.

## Concluding remarks

IL-33, a cytokine that is known to be involved in allergic diseases, plays diverse roles in the tumor immune system. Both the protumor and antitumor effects of IL-33 are contingent upon specific contexts and concentrations. The intricate interplay between IL-33 and various immune cells highlights its potential impact on tumor immunity. Low doses of endogenous IL-33 exert protumor effects by expanding Treg populations, promoting TAM M2 polarization, and directly inducing the proliferation or migration of tumor-prone cells. These effects collectively contribute to tumor progression, angiogenesis, and metastasis. On the other hand, higher doses of exogenous IL-33 can also promote Treg expansion, suppressive myeloid cell accumulation, and promote M2 polarization. However, high-dose exogeneous IL-33 overcomes these immunosuppressive effects by promoting antitumor responses through the activation of several antitumoral immune cells, including eosinophils, T cells, NK cells, and DCs. Given the potential of IL-33 as an adjuvant and a stimulator of DCs, IL-33 has emerged as a pivotal candidate for successful DC-based tumor immunotherapy. Although IL-33 can directly activate DCs and increase immunogenicity, several factors derived from IL-33-primed adjacent cells, such as basophils, induce the activation of immunogenic FCGR3^+^CD103^+^ cDC1s. These cDC1s exhibit superior immunogenicity and enhance CD8^+^ T-cell responses, leading to robust CTL responses and potent antitumor effects. In the realm of human DC-based tumor immunotherapy, IL-33 presents promising avenues. Direct IL-33 injection into cancer patients, generation of human immunogenic cDC1s from CD34^+^ hematopoietic progenitor cells using IL-33-derived factors, and development of highly immunogenic human Mo-DCs using IL-33-induced factors from human PBMCs are proposed strategies. These approaches aim to translate the observed antitumor effects of IL-33 in mice to effective DC-based tumor immunotherapy in humans.

In summary, IL-33 is a multifaceted cytokine with the potential to shape the tumor microenvironment. Understanding the delicate cooperation between IL-33 and DC-based antitumor effects provides valuable insights for tailoring therapeutic strategies harnessing IL-33 in the complex landscape of cancer immunotherapy.
